# Spatio-Temporal Interdependence of Bacteria and Phytoplankton during a Baltic Sea Spring Bloom

**DOI:** 10.3389/fmicb.2016.00517

**Published:** 2016-04-21

**Authors:** Carina Bunse, Mireia Bertos-Fortis, Ingrid Sassenhagen, Sirje Sildever, Conny Sjöqvist, Anna Godhe, Susanna Gross, Anke Kremp, Inga Lips, Nina Lundholm, Karin Rengefors, Josefin Sefbom, Jarone Pinhassi, Catherine Legrand

**Affiliations:** ^1^Centre for Ecology and Evolution in Microbial Model Systems - EEMiS, Linnaeus UniversityKalmar, Sweden; ^2^Aquatic Ecology, Lund UniversityLund, Sweden; ^3^Marine Systems Institute, Tallinn University of TechnologyTallinn, Estonia; ^4^Finnish Environmental Institute/Marine Research CentreHelsinki, Finland; ^5^Environmental and Marine Biology, Åbo Akademi UniversityÅbo, Finland; ^6^Department of Marine Sciences, University of GothenburgGothenburg, Sweden; ^7^Natural History Museum of Denmark, University of CopenhagenCopenhagen, Denmark

**Keywords:** 16S rRNA, marine bacteria, bacterioplankton, phytoplankton, *Skeletonema marinoi*, spring bloom, Baltic Sea, spatio-temporal

## Abstract

In temperate systems, phytoplankton spring blooms deplete inorganic nutrients and are major sources of organic matter for the microbial loop. In response to phytoplankton exudates and environmental factors, heterotrophic microbial communities are highly dynamic and change their abundance and composition both on spatial and temporal scales. Yet, most of our understanding about these processes comes from laboratory model organism studies, mesocosm experiments or single temporal transects. Spatial-temporal studies examining interactions of phytoplankton blooms and bacterioplankton community composition and function, though being highly informative, are scarce. In this study, pelagic microbial community dynamics (bacteria and phytoplankton) and environmental variables were monitored during a spring bloom across the Baltic Proper (two cruises between North Germany to Gulf of Finland). To test to what extent bacterioplankton community composition relates to the spring bloom, we used next generation amplicon sequencing of the 16S rRNA gene, phytoplankton diversity analysis based on microscopy counts and population genotyping of the dominating diatom *Skeletonema marinoi*. Several phytoplankton bloom related and environmental variables were identified to influence bacterial community composition. Members of *Bacteroidetes* and *Alphaproteobacteria* dominated the bacterial community composition but the bacterial groups showed no apparent correlation with direct bloom related variables. The less abundant bacterial phyla *Actinobacteria, Planctomycetes,* and *Verrucomicrobia,* on the other hand, were strongly associated with phytoplankton biomass, diatom:dinoflagellate ratio, and colored dissolved organic matter (cDOM). Many bacterial operational taxonomic units (OTUs) showed high niche specificities. For example, particular *Bacteroidetes* OTUs were associated with two distinct genetic clusters of *S. marinoi*. Our study revealed the complexity of interactions of bacterial taxa with inter- and intraspecific genetic variation in phytoplankton. Overall, our findings imply that biotic and abiotic factors during spring bloom influence bacterial community dynamics in a hierarchical manner.

## Introduction

In the brackish Baltic Sea, the phytoplankton spring bloom generally begins in coastal areas and propagates toward the central parts of the basins. The timing of the onset is slightly lagged in the northern parts compared to the southern Baltic (Godhe et al., 2016). Dinoflagellates and diatoms dominate the spring bloom (Wasmund et al., 1998) and the model diatom species *Skeletonema marinoi* accounts for up to 10^4^ cells per ml in the Kattegat during spring (Saravanan and Godhe, 2010). *Skeletonema marinoi* forms two distinct genetic populations in the Baltic Sea, one mainly dominating in the southern Baltic and the other being predominant in the middle and northern Baltic Proper (Sjöqvist et al., 2015; Godhe et al., 2016). The genetic population structure of *S*. *marinoi* is influenced by oceanographic dispersal barriers and the salinity regime, similar to population structures of other marine organisms (Jørgensen et al., 2005; Johannesson and Andre, 2006). The Baltic Sea salinity gradient also influences the bacterioplankton community composition during summer (Herlemann et al., 2011; Dupont et al., 2014). However, the bacterioplankton community composition during the spring bloom has to our knowledge not been assessed in a spatial-temporal survey covering the entire Baltic Proper. In addition, knowledge about how spring phytoplankton populations structure co-occurring bacteria in the Baltic Sea is still limited.

The primary production of the spring bloom in the Baltic Sea exceeds production estimates of the summer cyanobacterial bloom (Legrand et al., 2015) and the organic matter produced during the spring bloom is a main source for bacterial production (Lindh et al., 2014). Bacterial taxa differ in their capabilities to degrade organic carbon compounds (Gómez-Consarnau et al., 2012) and especially *Flavobacteria* are reported to utilize high molecular organic matter released from phytoplankton blooms (Buchan et al., 2014). Therefore, the bacterial community composition during spring, commonly dominated by *Bacteroidetes, Alphaproteobacteria,* and *Actinobacteria* (Andersson et al., 2010; Lindh et al., 2014), might also indirectly be influenced by environmental variables that structure the phytoplankton spring bloom. So far, studies focusing on bacterial communities accompanying and interacting with phytoplankton blooms have been mostly carried out in limnic systems or laboratory mesocosm experiments (Bell and Lang, 1974; Cole, 1982; Riemann et al., 2000; Pinhassi et al., 2004; Fandino et al., 2005; Teeling et al., 2012; Buchan et al., 2014).

This study aimed to assess the bacterioplankton community composition during the Baltic Sea spring bloom. We studied how bacterial groups interacted with phytoplankton phyla and which bacteria co-occurred with specific populations of the diatom *S. marinoi*. Further, we identified the correlations of marine bacteria to environmental and bloom related variables, such as phytoplankton biomass.

## Materials and methods

### Sampling and environmental variables

**Figure 1:**
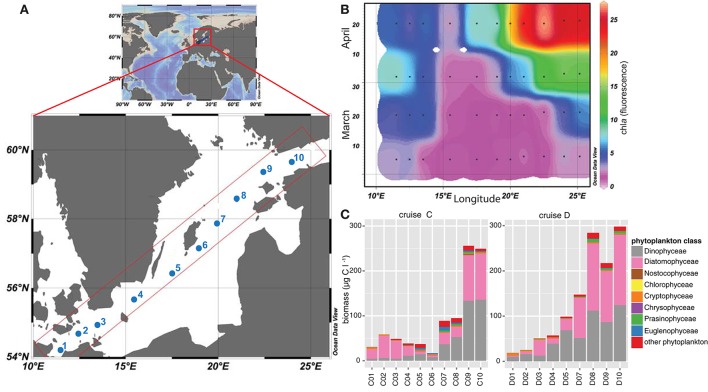
**Map of the Baltic Sea illustrating the sampling stations included in this study. (A)** The map demonstrates the Baltic Sea, within Europe, and the sampling transect over the Baltic Proper. Sampling stations during the four cruises (A–D) are labeled from 1 to 10, ranging from stations 1-3 in the southern and stations 8–10 in the northern coastal part of the Baltic Proper. Stations 4-7 were located in the open Baltic Proper. **(B)** Relative chl*a* fluorescence [measured with the flow through system on board and modified from Godhe et al. (2016)] across the transects over the Baltic Proper from March to April 2013 (cruise A–D). Black dots describe sampling occasions, values were extrapolated using Ocean Data View. **(C)** Phytoplankton biomass in μg C l^−1^ during cruise C (early April) and D (late April) along the transect, based on microscopy counts.

To analyze the phytoplankton spring bloom succession and its consequences, surface water samples (8 m depth) were collected during four research cruises [Cruise A (4th–7th March), B (19th–22nd March), C (4th–7th April), and D (16th–19th April)] during spring 2013. We used the Alg@line facilities on board the ship of opportunity MS *Finnmaid*, organized by SYKE (Finnish Environment Institute) and PRODIVERSA (Population genetics and intraspecific diversity of aquatic protists across habitats and eucaryotic clades, NordForsk Researcher Network). Ten stations were sampled along a northeast to southwest transect across the Baltic Proper from the Gulf of Finland to the southern Baltic Proper. Environmental variables and chlorophyll *a* (measured by relative chl*a* fluorescence, as a proxy for phytoplankton biomass) were measured using a ferrybox measurement system connected to a flow through system onboard. More specifically, the ferrybox is an automated system that measures chlorophyll fluorescence, temperature, salinity, and cDOM fluorescence while the ship is moving (Rantajärvi, 2003). Nutrients (nitrate, phosphate, silicate) were automatically collected on board utilizing an automated sample carousel containing 24 bottles, and were analyzed at SYKE using methods as described in Grasshoff et al. (1983) and Godhe et al. (2016). The map of the Baltic Sea and chl*a* values (Figure 1) were drawn with Ocean Data View 4 (Schlitzer, 2014).

### Phytoplankton counts and *s. marinoi* genotypying

Phytoplankton samples were fixed with acidic Lugol onboard and were counted using a light microscope (LEICA DM IL Bio, GF10/18M Ocular, 200x or 400x magnification). Water samples from each station (25 ml sample water) were sedimented for 24 h in a sedimentation chamber (26 mm diameter), HELCOM biovolume guidelines (Olenina et al., 2006) were followed and carbon concentrations were estimated, and are presented in Figure 1C.

*S. marinoi* strains were genotyped using eight microsatellite loci (Almany et al., 2009), and assigned to populations using a Bayesian structure analysis using the software STRUCTURE [cluster membership (*K* = 2); Pritchard et al., 2000], based on the microsatellite data as previously reported (Figure S1 in Godhe et al., 2016).

### Bacterial analyses

Samples for bacterial abundance were fixed in duplicates with formalin (3% final concentration, Sigma-Aldrich) and stored at −20°C until processing. Subsamples were stained with SYTO®-13, a green fluorescence nucleic acid stain (Life technologies^TM^), normalized with truecount beads and counted with a flow cytometer (FACScalibur). Bacterial abundance data were averaged for technical duplicates, bacterial counts and standard deviations are provided in Supplementary Table 1.

Samples for bacterial biomass were obtained during cruises C (five stations) and D (nine stations) in April 2013. Water samples for DNA extraction (1 L) were filtered on 0.2 μm supor® membrane filters (PALL Life Sciences) and preserved in TE-buffer (Tris-EDTA Buffer, Sigma® Life Science) at −20°C. DNA was extracted using a phenol/chloroform protocol adapted from Boström et al. (2004). In brief, lysozyme was added to the samples (1.1 mg ml^−1^ final concentration in TE buffer) and incubated at 37°C for 30 min. SDS and proteinase K were added to the samples (final concentration 1%, and 0.1 mg ml^−1^, respectively) and incubated at 55°C overnight. Phenol/chloroform/isoamyl alcohol (25:24:1) was added in equal volumes prior to transfer of the water phase, before the samples were washed with chloroform/isoamyl alcohol (24:1). This phenol extraction step was repeated twice before sodium acetate was added (1/10 volume) and DNA was precipitated with 100% ethanol (equal amounts) before centrifugation to receive a DNA pellet. The DNA pellet was air dried and dissolved in 1x TE buffer.

The V3–V5 region of the 16S rRNA gene was amplified using the primers 341F and 805R by Herlemann et al. (2011), as previously described (Lindh et al., 2015). Briefly, two PCRs were conducted per sample. The first PCR amplified the 16S rRNA gene and added Illumina adapters [per reaction: 1.25 μl F/R primer-Illumina adapter at 10 pM, 0.5 μl DNA template, 12.5 μl Phusion Mastermix (ThermoScientific) and 9.5 μl H_2_O] in 20 PCR cycles [98°C 30 s, (98°C 10 s/58°C 30 s/72°C 15 s) × 20 cycles, 72°C 2 min]. After cleaning the PCR1 product using AmpPureXP following the manufacturer's instructions, the second PCR served to attach standard Illumina handles and Illumina index primers. PCR conditions [per reaction 11.5 μl cleaned PCR1 product, 12.5 μl Phusion Mastermix (ThermoScientific) and 0.5 μl F/R primer] included 12 cycles [98°C 30 s, (98°C 10 s/62°C 30 s/72°C 5 s) × 12 cycles, 72°C 2 min]. DNA concentrations of PCR products were measured using a Qubit 2.0 fluorometer (Invitrogen) and quality controlled for appropriate fragment length on an agarose gel. Samples were pooled and sequenced on a Miseq Illumina platform (2 × 300 bp) at Scilife (SciLifeLab, Stockholm). DNA sequences have been deposited at the NCBI Sequence Read Archive under project number PRJNA308537.

### Bioinformatics and statistical analyses

To analyze factors that structure the bacterial community composition, stations C1:C3, C9:C10, D1:D5, and D7:D10 were sampled simultaneously with the phytoplankton bloom and environmental variables. Illumina 16S rRNA gene sequences were analyzed according to the Uparse pipeline (Edgar, 2013). In short, sequences were stripped, merged, and quality filtered according to default settings (Edgar, 2013). The total number of sequences obtained from the Illumina platform was 1.6 million sequences. After merging the sequences (0.9 million r1-sequences) 82% passed quality control, resulting in 700,474 total sequences with an average read length of 453 bp. Sequences that passed quality control were sorted and clustered using a radius of 1.5%, resulting in 97% sequence identity, 586,308 sequences were obtained after quality control. Reads were annotated using a basic local alignment tool (BLAST) against the SILVA database SSURef99 release 119 (downloaded 14th June 2014), using the SINA aligner (Altschul et al., 1990; Pruesse et al., 2012). Clustering and annotation of the sequences resulted in 45.6% chloroplast reads. On average, 25,127 sequences were obtained per sample after excluding chloroplast sequences. Three bacterial OTUs of the 50 most abundant OTUs in the dataset were annotated as “bacteria” in the SILVA database (OTU_0016, OTU_0031, OTU_0040). An additional search in the NCBI database (NCBI Resource Coordinators, 2013; 29th February 2016) resulted in ≤92% sequence similarity to actinobacterial sequences, thus the OTUs are referred to as “other bacteria.”

Bacterial community analyses and statistical analysis were conducted in RStudio Version 0.98.1103 using the packages vegan, ggplot2, dplyr, ComplexHeatmap, pls, gridExtra, and RColorBrewer (Gentleman et al., 2004; Oksanen et al., 2007; Wickham, 2009; Neuwirth, 2014; Auguie, 2015; Gu, 2015; Mevik et al., 2015; Wickham and Francois, 2015). The bacterioplankton community is hereafter referred to as annotated OTUs excluding chloroplast reads. To obtain relative OTU abundances, the OTU reads were normalized using a total-sum normalization and are presented as % of total abundance per sample.

Circos graphs for bacterial community compositions were drawn using the online circos software (Krzywinski et al., 2009). Betadiversity measurements were based on Bray-Curtis dissimilarity matrices (Bray and Curtis, 1957). Partial least squares (PLS) regressions was performed by using the PLS package in R (Mevik et al., 2015). Correlations between bacterial families and chemical and biological variables, as well as between the top 50 OTUs and chemical and biological variables were generated using Pearson correlations.

## Results

### Environmental variables and phytoplankton bloom dynamics

On the route between Finland and Germany (Figure 1A), salinity ranged from 5.5 PSU in the northernmost part of the transect to 10.2 PSU in the South (Supplementary Table 1). Temperature ranged from 2.8°C in the southernmost station (station C1) to 1.4°C and ice cover in the northernmost station (station C10) in early April (4th–7th April), and 5.8 to 2.3°C in late April (16th–19th April, Supplementary Table 1). The spring bloom successively depleted the inorganic nutrients along the entire transects. Concentrations of nitrate and nitrite were low in late April, on average 0.03 μM. Silica was high at the northernmost station (station D10; 16.7 μM) during the first sampling, and still measured 11.6 μM in the second half of April at the Gotland Deep (station D5) and up to 10.1 μM in the north (station D10). At the southernmost station (station D1), the silica level had dropped to 0.1 μM. Phosphate concentrations in late April ranged between values below detection limit and 0.3 μM (Supplementary Table 1). Chl*a* fluorescence followed the spring bloom progression and increased constantly from March until late April, and ranged from 1.7 at the Gotland Deep (station C5) up to 27.0 at the northern stations (station D9; Figure 1B). Station 4, close to Bornholm Deep, exhibited the lowest chl*a* values, but increased from 1.9 to 2.7 during April.

Phytoplankton biomass (based on microscopy counts excluding ciliates) was significantly lower in the sea area south of the Gotland Deep (station 1–4) compared with values obtained for the Northern Baltic Proper and Gulf of Finland during both cruises in April (station 9–10; Figure 1C). Phytoplankton biomass ranged from 30.8 μg C l^−1^ at the southern stations to 16.9 μg C l^−1^ at station C6 in the Baltic Proper, and up to 297 μg C l^−1^ at station in the north (station D10). Diatoms and dinoflagellates dominated the spring bloom with on-average 43 and 41% of the total phytoplankton biomass, respectively (Figure 1C). Dinoflagellates displayed a pronounced bloom at stations 9 and 10 during both cruises with biomass up to 133.6 μg C l^−1^. Among the southern stations, diatom biomass peaked at 49 μg C l^−1^ at station C2, while among the northern stations, diatom biomass peaked at 156 μg C l^−1^ at station D10. The ratio between diatom and dinoflagellate biomass, as a proxy for phytoplankton community composition, was higher in the southern stations, emphasizing the dominance of diatoms in these areas. Other phytoplankton classes, e.g., *Euglenophyceae, Cryptophyceae,* and *Nostocophyceae* were identified, but biomass of these phyla did not exceed 10 μg C l^−1^.

The diatom *S. marinoi* accounted for on average 5.88% (*sd* = 7.39%) of total phytoplankton biomass (data not shown) and exhibited two genetic clusters (cluster 1 and cluster 2), as defined in a related study (Godhe et al., 2016) and reprinted in Supplementary Table 1. Cluster 1 dominated stations C1–C3, C9–C10, and D1–D4, whereas cluster 2 dominated stations D5–D10.

### Bacterial abundance and community composition

Bacterial abundance ranged between 9 × 10^5^ cells ml^−1^ at stations 9 and 10 to 1.7 × 10^6^ cells ml^−1^ at stations 1–3 during cruise C and reached a maximum of 2.8 × 10^6^ cells ml^−1^ during cruise D.

**Figure 2:**
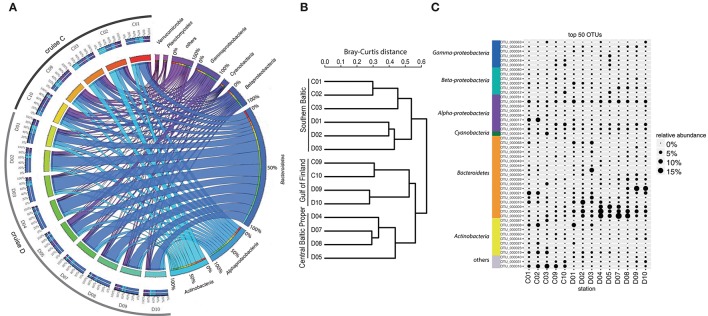
**Bacterial community composition and most abundant OTUs**. **(A)** The figure displays relative OTU abundances grouped by bacteria phyla/classes in a circus graph. The right side of the graph visualizes relative OTU abundances of major bacteria phyla/classes summed over cruises C and D, as indicated by the size of the cells for each taxon (note color coding of ribbons connecting taxa with stations). Also shown are axes/cells for the taxon-normalized abundances at each station, as color-coded by station in the left side of the graph. Ribbons connect the relative abundances of the taxa with the color-coded stations on the left side of the graph, and width of ribbons is proportional to relative abundance. The left side of the graph indicates the relative abundances of the major taxa (color-coded according to taxa on right side of graph) at each sampled station during cruises C and D. Color-coding of stations (from red to blue-green) identifies proportions in inner circle/cells on right side of graph. **(B)** Beta-diversity analysis (beta-dendrogram, based on Bray–Curtis distance matrix), and **(C)** relative abundance patterns of the 50 most abundant OTUs across all stations. The group “others” in panel C comprises OTUs that have ≤92% sequence similarities to known sequences in NCBI (NCBI Resource Coordinators, 2013). Relative abundance is calculated as % of total sequences obtained per sample (excluding sequences annotated as chloroplasts).

Analysis of bacterioplankton community composition (excluding chloroplasts) showed that *Actinobacteria* (13.5%), *Alphaproteobacteria* (15.4%), and *Bacteroidetes* (42.4%) dominated the bacterial community during the spring bloom (Figure 2A). Beta-diversity analysis of all normalized sequences grouped the bacterial communities into three clusters (Figure 2B). The first cluster comprised the southern stations 1, 2 and 3. The second cluster grouped the middle stations 4, 5, 7, and 8 while the third cluster grouped stations 9 and 10 in the Gulf of Finland together.

The most abundant bacterial OTUs (top 50 OTUs) displayed pronounced differences in spatial-temporal distribution patterns (Figure 2C). Some OTUs showed enhanced relative abundances in the middle of the Baltic Proper; for example OTU_0002 (*Flavobacteriaceae*) accounted for on average 5.9% of the relative bacterial abundance per station and peaked at station D07 (Figure 2C). Other OTUs increased in relative abundance with time, OTU_0022 (*Polaribacter*) showed the highest relative abundance right after the chl*a* peak in the northernmost stations. Some OTUs bloomed in the southernmost stations (e.g., OTU_0017, *Rhodobacteriaceae*), while OTU_0016 (other bacteria) showed higher relative abundances in early April compared to late April.

### Bacterial community linked to environmental variables and phytoplankton groups

**Figure 3:**
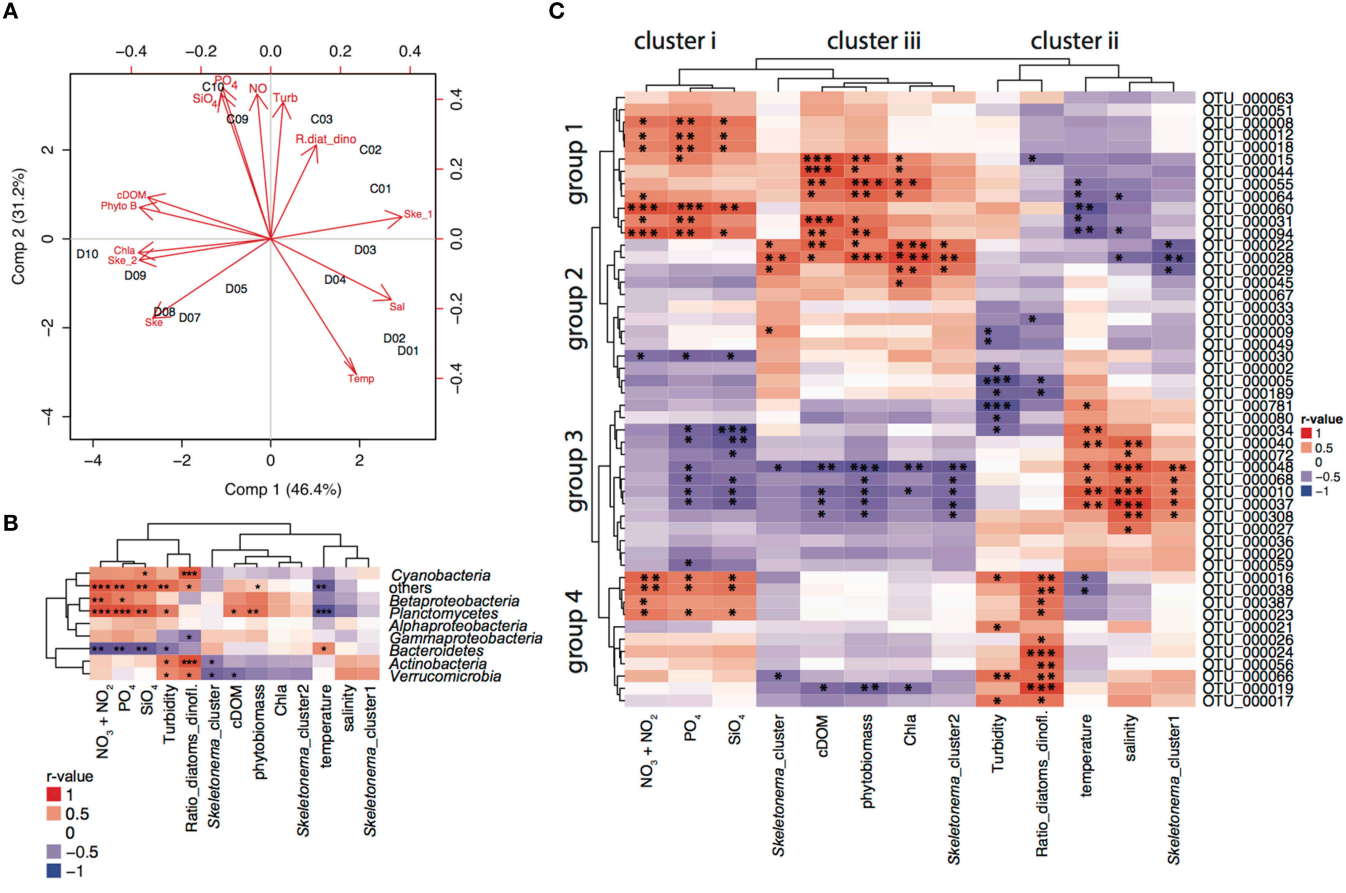
**PLSr and correlations to environmental variables. (A)** Partial least squares regression analysis (PLSr) linking bacterial community composition (including all OTUs) with environmental variables and biotic factors in stations 1–3, 9–10 during cruise C, and 1–10 during cruise D. Red short labels describe the environmental variables (PO_4_; phosphate, NO; nitrate and nitrite, SiO_4_; silicate, Turb; turbidity, R.diat_dino; diatom/dinoflagelate ratio, cDOM; colored fluorescent DOM, Phyto B; phytoplankton biomass, Chla; chlorophyll a fluorescence, Ske_2; S. marinoi cluster 2, Ske; S. marinoi cluster, Temp; temperature, Sal; salinity, Ske_1; S. marinoi cluster 1) whereas black labels describe the sampling stations. **(B)** Correlation analysis between relative abundances of bacterial families and environmental variables based on Pearson correlations for relative abundances of bacterial families and environmental variables. Correlation values depict *r*-values of Pearson correlations and the dendograms are based on Pearson correlations. Statistical significance levels: *p < 0.05, **p < 0.01, ***p < 0.001. **(C)** Correlation analysis between relative abundances of 50 most abundant OTUs and environmental variables based on Pearson correlations. Correlation values depict *r*-values of Pearson correlations. Statistical significance levels: *p < 0.05, **p < 0.01, ***p < 0.001.

Partial least squares regression analysis (PLSr analysis) identified the cluster of environmental variables shaping bacterial community composition depending on the sampling location (Figure 3A). In the PLSr, the first and second component explained 77.6% of the bacterioplankton community structure variability. No clear pattern of community structure could be detected with salinity and latitude gradients. While higher levels of nutrients, turbidity and ratio of diatoms:dinoflagellates were linked to communities sampled in cruise C, high temperatures were associated to bacterioplankton during cruise D. Northern stations assemblages were related to high cDOM, phytoplankton biomass, chl*a*, and *S. marinoi* cluster 1. On the other hand, *S. marinoi* cluster 2 was tightened to southern station communities. To further disentangle the relationship between environmental variables and bacteria families and individual OTUs, we conducted correlation analysis.

Pearson correlations of bacterial families to biotic and abiotic factors revealed that *Bacteroidetes* were positively correlated to temperature and negatively correlated to nutrient concentrations [nitrate (*p* = 0.001), phosphate (*p* = 0.003), silicate (*p* = 0.005), temperature (*p* = 0.040); Figure 3B]. *Planctomycetes* were most prevalent in the Gulf of Finland (stations 9 and 10) and were positively correlated with phytoplankton biomass (*p* = 0.009), cDOM (*p* = 0.016), and nutrients [nitrate (*p* = 0.000), phosphate (*p* = 0.000), silicate (*p* = 0.002)]. Diatom:dinoflagellate ratios positively influenced *Cyanobacteria* (*p* = 0.001), *Actinobacteria* (*p* = 0.000), and *Verrucomicrobia* (*p* = 0.015). Furthermore, *Actinobacteria* (*p* = 0.045) and *Verrucomicrobia* (*p* = 0.014) correlated significantly with the genetic population structure of *S. marinoi*. *Betaproteobacteria* linked to nutrient concentrations [(nitrate (*p* = 0.003), phosphate (*p* = 0.020), silicate (*p* = 0.075)], while relative abundances of *Alphaproteobacteria* could not be explained by any measured environmental variables.

The relative abundance patterns of the 50 most abundant OTUs (top 50 OTUs) correlated differently to the measured environmental variables (Pearson correlation, Figure 3C). The environmental and bloom related variables could be separated into three clusters according to how they correlated to the top 50 OTUs: (i) nutrients (nitrate, phosphate, and silicate); (ii) salinity, turbidity, diatoms/dinoflagellates, temperature, *S. marinoi* cluster 1; and (iii) chl*a*, cDOM, phytoplankton biomass, *S. marinoi* cluster 2. The first group of the most abundant OTUs was positively associated to cluster i and iii, and negatively correlated to cluster ii and consisted of a variety of taxa. The second group consisted of mostly *Bacteroidetes* and *Alphaproteobacteria*, but excluded *Actinobacteria* and was only positively correlated to cluster iii. The third group mainly consisted of *Flavobacteria* and *Actinobacteria* that were only positively linked to cluster ii while negatively correlated to cluster i and iii. Lastly, the fourth group included three OTUs of the *Actinobacteria*, three *Alphaproteobacteria* OTUs, two *Flavobacteria* and one *Betaproteobacteria* OTU and was largely positively correlated to cluster i, and the variables in cluster ii, and negatively to the variables in cluster iii. Furthermore, four *Bacteroidetes* populations (OTU_0056, OTU_0024, and OTU_0026) were only positively correlated to diatom:dinoflagellate ratio, whereas several OTUs of groups 1 and 2 showed strong correlations with phytoplankton biomass. OTU_0022 (*Polaribacter*) was strongly correlated to chl*a, S. marinoi* cluster 2 and phytoplankton biomass (Figure 3C). OTU_0189 (*Pseudorhodobacter*) was ubiquitously found at all stations but was still negatively correlated to turbidity and diatom:dinoflagellate ratio. *S. marinoi* cluster 1 impacted strongly on several co-occuring *flavobacteria* (OTU_0048, OTU_0068, OTU_0010), *Comamonadaceae* BAL58, and one OTU annotated as *Candidatus aquiluna*.

### OTUs uniquely associated to *S. marinoi* populations

**Figure 4:**
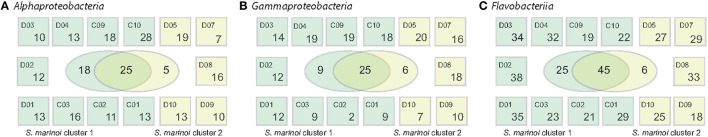
**Spatial distribution of OTUs across the Baltic Sea. (A)** *Alphaproteobacteria*, **(B)** *Gammaproteobacteria*, and **(C)** *Flavobacteriia*. The Venn diagrams in the center of each panel depict the number of OTUs unique or shared between the stations where *S. marinoi* cluster 1 (dark green) or *S. marinoi* cluster 2 (light green) dominated. Small boxes depict how many OTUs of each major taxon that were detected at each station (numbers include OTUs that occur at single or multiple stations). Only OTUs with relative abundances >0.1% are included.

Within the *Alphaproteobacteria, Gammaproteobacteria,* and the *Flavobacteriia* family, numerous OTUs were commonly found at several stations during April, comprising >0.1% of relative abundances (Figure 4). To investigate if these showed unique abundance patterns correlating with the different genetic populations of *S*. *marinoi*, the sampling stations were grouped according to the dominant *S. marinoi* genetic cluster at each station. Twenty-five *Alphaproteobacteria* OTUs were shared among the two diatom populations, while 18 OTUs were uniquely found at stations where *S. marinoi* cluster 1 dominated. Three of these OTUs reached relative abundances >5% of the bacterial community. Of these, *Rhodobacteriaceae* (OTU_0017) and a member of the SAR11 clade annotated as Chesapeake-Delaware Bay (OTU_0015) co-occurred with *S.marinoi* cluster 1. *Pseudorhodobacter* (OTU_0189) on the other hand exhibited high abundances (>5%) during the second half of April at stations D1, D7, and D10, and was not uniquely associated with any *S. marinoi* cluster. Among *Gammaproteobacteria*, 25 OTUs occurred with both *S. marinoi* populations, nine OTUs were associated with station where *S. marinoi* cluster 1 dominated, and six OTUs were exclusive to the dominating *S. marinoi* cluster 2 found in the northern Baltic during cruise D. None of the gammaproteobacterial OTUs accounted for relative abundances >5%. Interestingly, *Flavobacteriia* showed an even stronger trend compared to the *Proteobacteria*, and 45 OTUs were shared between stations dominated by ether genetic clusters while 25 flavobacteriial OTUs were uniquely found at stations with a dominance of *S. marinoi* cluster 1. Four of the OTUs unique to *S. marinoi* cluster 1 accounted for >5% of the bacterial abundance (OTU_0036, OTU_0010, OTU_0068, OTU_0026). Three of the highly abundant OTUs were shared while *Polaribacter* (OTU_0022) was uniquely found at stations dominated by *S. marinoi* cluster 2.

## Discussion

In the present study we assessed the microbial community composition and co-occurrences on a spatio-temporal gradient during a Baltic Sea phytoplankton spring bloom. This analysis uncovered that bacterial community composition in the surface waters, both at the level of major families and at the level of individual populations (OTUs), was strongly correlated to various environmental variables. Most notably, bacterial community structure was associated with a number of variables directly related to phytoplankton, i.e., measures of chl*a*, phytoplankton biomass, diatom:dinoflagellate ratio, and *S. marinoi* population clusters. Secondly, the distribution of bacteria was also correlated with variables indirectly influenced by phytoplankton, including nutrient concentrations and DOM. Physicochemical variables, such as temperature and salinity also had additional but minor effects on bacterioplankton dynamics. We thus suggest that biotic and abiotic factors during spring bloom influence spatio-temporal bacterioplankton dynamics in a hierarchical manner.

The southern areas of the Baltic Sea are influenced by saline water inflows from the North Sea, and salinity decreases northwards. This strongly controls the distribution of phytoplankton in the Baltic Sea. For the diatom *S. marinoi*, which is typically a dominant component of Baltic Sea spring blooms, oceanographic connectivity and salinity are key determinants of population dynamics in space and time (Sjöqvist et al., 2015; Godhe et al., 2016). Salinity is a recognized overall driver also affecting the spatial distribution of bacterial populations in surface waters in the Baltic Sea, as shown for 16S rRNA gene amplicon and metagenome studies (Herlemann et al., 2011; Dupont et al., 2014). These spatial studies were undertaken during summer when environmental conditions are relatively stable in stratified surface waters. Laboratory experiments have shown a similar trend, in that salinity can act as a selective force for bacterial community compositions (Langenheder et al., 2003; Kaartokallio et al., 2005). During our spring study on the other hand, salinity and oceanic connectivity varied minimally over time, although the salinity gradient affected bacteria at the spatial scale (Gulf of Finland and southern Baltic). Therefore, the observed changes in bacterioplankton community composition over time indicated that salinity (or temperature) were minor determinants shaping bacterial temporal dynamics. This substantiated that phytoplankton played a major role for structuring bacterioplankton composition.

Various seasonal studies and laboratory mesocosm experiments have established that peaks in phytoplankton blooms are followed by elevated bacterial production and abundance (see for example: Cole, 1982; Brussaard et al., 1996; Riemann et al., 2000; Pinhassi et al., 2004; Lindh et al., 2014). In our study, bacterial community composition was linked to phytoplankton biomass and chl*a*, whereas bacterial abundance was not. Though phytoplankton biomass was higher in the Gulf of Finland, bacteria did not show as high abundance as in the south, possibly due to lower temperature. Diatom:dinoflagellate ratio is a measure for phytoplankton community composition and succession of the bloom and correlated to the bacterioplankton community composition. Different phytoplankton groups are commonly accompanied by specific bacterial taxa (Pinhassi et al., 2004; Amin et al., 2012; Buchan et al., 2014). Diatoms are often associated with *Alphaproteobacteria* and *Bacteroidetes* (Amin et al., 2012), while mainly *Flavobacteria* (*Bacteroidetes*) have been shown to co-occur with the particle fraction of a dinoflagellate bloom (Fandino et al., 2001). At the family level of resolution, *Bacteroidetes* did not show significant correlations to phytoplankton biomass or the diatom:dinoflagellate ratio. Still, among the most abundant specific bacterial populations, such associations were observed. For example, three bacterial populations (*Bacteroidetes, Alphaproteobacteria, Cyanobacteria*) showed preferences for only high diatom:dinoflagellate ratios, whereas two *Bacteroidetes* OTUs were associated with more even ratios. Notably, those bacteria were not correlated to phytoplankton biomass or chl*a*, but mainly to phytoplankton groups. These findings emphasize the importance of phytoplankton species composition, not solely phytoplankton biomass, for determining bacterioplankton community composition.

During the spring bloom, the contrasting influence of oceanic and coastal factors is recognized to determine the distribution of the two genotypically distinct populations of the diatom *S. marinoi* population (denoted cluster 1 and 2; Godhe et al., 2016). The bloom in the southern Baltic was dominated by *S. marinoi* cluster 1, influenced by mainly oceanic features like higher salinity and temperature and lower chl*a*. Accompanying this diatom cluster was a higher richness of *Alphaproteobacteria, Gammaproteobacteria,* and *Flavobacteriia*. This might result in a high diversification of metabolic strategies of bacterioplankton feeding on the wide spectrum of DOM compounds so as not to waste valuable, good quality DOM and their associated bacterioplankton communities.

*Skeletonema marinoi* cluster 2 was accompanied by coastal features such as cDOM from land runoff, exceeding 50% of marine DOM in the Baltic Sea (Deutsch et al., 2012), low temperatures due to late ice melt, and relatively high nutrients and chl*a.* Bacteria associated with *S. marinoi* cluster 2 therefore likely benefit from both phytoplankton DOM and terrestrial DOM. *Flavobacteriia, Alphaproteobacteria,* and *Gammaproteobacteria* showed a lower richness at stations where *S. marinoi* cluster 2 dominated the diatom population. This lower richness could be due to a slight delay of the bacteria responding to the bloom and diatom cluster 2 and low temperatures, but since *S. marinoi* strains co-occur in all stations, this does not seem likely. A delayed response in bacterial richness, commonly observed during phytoplankton blooms (Buchan et al., 2014), seems instead to be linked to gene cluster dominance and not to the succession of gene clusters.

Our study revealed a potential influence of phytoplankton genotypes on bacterial populations. Accordingly, several bacterial OTUs showed distinct distributions associated to *S. marinoi* genotypes. A number of papers indicate direct interactions between phytoplankton and bacteria (Delucca and McCracken, 1977; Amin et al., 2015; Durham et al., 2015) and point to molecular mechanisms as possible explanations for the tight correlations observed in this study. Overall correlations of major phytoplankton and bacterial groups, and additionally more taxonomically restricted relations support the hypothesis that such interactions are important in structuring microbial communities in the sea.

Phytoplankton indirectly affects DOM concentrations by using up available nutrients and producing organic molecules during photosynthesis. Commonly, phytoplankton blooms release up to 20% of their daily primary production as dissolved organic matter (DOM) into the water (Baines and Pace, 1991; Wear et al., 2015), and create an environment with niches that are exploited by numerous, opportunistic bacteria (Bell and Lang, 1974; Gilbert et al., 2012; Martin, 2012; Teeling et al., 2012; Needham et al., 2013). Marine bacteria utilize various DOM compounds, leading to complex community dynamics (composition, taxa abundance and richness) during the duration of phytoplankton blooms (Riemann et al., 2000; Pinhassi et al., 2004; Buchan et al., 2014). In our study, high abundance of opportunistic *Flavobacteriia* and an increase in *Alphaproteobacteria* were observed during late April, coinciding with a detected depletion of inorganic nutrients. Thus, it appears that inorganic nutrient concentrations, together with an increase of DOM, fuelled bacterial growth and affected bacterial community composition. Increases in DOM lead to remineralization of nutrients and possibly prolong the growth phase of both phytoplankton and bacterioplankton, which in turn can increase the activity of the microbial loop.

In offshore waters of the central Baltic Sea, the bacterial community composition was distinct in comparison to the ones from the coastal basins (Gulf of Finland and southern Baltic Proper, Figure 2B). The presence of three highly abundant *Bacteroidetes* populations (OTU_0009, OTU_0002, OTU_0005) associated with low turbidity, but not phytoplankton biomass. This suggests that this community dominated by *Bacteroidetes* could be a relict from overwintering, or characteristic of a bacterial community composition during early spring bloom initiation. Unfortunately, our data timeline (March–April) did not include these bloom stages in the coastal areas, hence limiting the comparison. However, the coastal to offshore gradient in the Baltic Sea, although small compared to oceanic province, still is relevant to explain the succession of microbial populations, including phytoplankton and bacterioplankton.

The contribution of *Actinobacteria* to the total bacterial community in the Gulf of Finland, as compared to *Flavobacteria*, was lower than expected. *Actinobacteria* commonly display high abundances in low salinity habitats of the Baltic Sea (Herlemann et al., 2011; Dupont et al., 2014). This can partly be explained by the increased contribution of *Flavobacteria* to the bacterial community composition following the bloom. *Flavobacteria* and other bacterial groups may be better or faster at using phytoplankton exudates. In fact, a recent study reported that *Actinobacteria* from the Baltic Sea seem to use lipids, rather than carbohydrates as a carbon source and are found more abundantly during the second half of the year and not during spring (Hugerth et al., 2015). The Luna bacteria, a subgroup of *Actinobacteria*, coincided with phytoplankton blooms, though these were different populations with distinctive metabolic features compared to the summer group (Hugerth et al., 2015). Moreover, one *Polaribacter* population reoccurs at high abundance during spring in the Gulf of Finland (Laas et al., 2015), indicating that this taxon is highly linked to coastal conditions. However, the relative abundance of this taxon is lower during other times of the year (Laas et al., 2015). In our study, a *Polaribacter* population was highly abundant in the Gulf of Finland and correlated to several coastal features, especially chl*a*, illustrating a tight coupling to spring phytoplankton biomass and possibly dinoflagellates. Consequently, synergistic effects of multiple direct and indirect bloom related factors, likely influence actinobacterial and overall bacterioplankton dynamics during spring.

## Conclusions

Our study revealed a complex array of interactions and interdependence of bacterial populations with intra-specific diversity of phytoplankton groups. Variables related to the spring bloom progression were of principal importance for determining bacterioplankton composition. In the Baltic Sea, oceanographic connectivity and salinity shape two different *S. marinoi* populations co-occurring with distinct bacterial communities. In large spring blooms, interactions between bacteria with distinct niches and specific phytoplankton populations may have implications for biomass production and cycling of energy to higher trophic levels. Altogether, our novel findings imply that biotic and abiotic factors during spring bloom influence overall bacterial community dynamics in a hierarchical manner.

## Author contributions

AG, KR, AK, CL, and NL conceived the study, CB and CL designed research, CB, MB-F, CL, CS, JS, NL, and SG performed sampling, CB and MB-F performed molecular analysis, SS and IL counted phytoplankton samples, CB, MB-F, IS, CS, JP, and CL analyzed data, CB, MB-F, JP, and CL wrote the paper. All authors discussed the results and commented on the manuscript.

## Funding

The study was funded by grants from the Nordforsk research network (PRODIVERSA), the Swedish Research Council Formas through the Strong Research Environment ECOCHANGE to CL and JP, institutional research funding (IUT 19-6) of the Estonian Ministry of Education and Research, and the Centre for Ecology and Evolution in Microbial Model Systems (EEMiS) at Linnaeus University. AK and CS received funding from the Academy of Finland grants 283061 and 251564.

### Conflict of interest statement

The authors declare that the research was conducted in the absence of any commercial or financial relationships that could be construed as a potential conflict of interest.
