# Supplementary material for: Spatio-Temporal Interdependence of Bacteria and Phytoplankton during a Baltic Sea Spring Bloom
Source: Front Microbiol. 2016 Apr 21;7:517. doi: 10.3389/fmicb.2016.00517 (PMC4838809; doi:10.3389/fmicb.2016.00517)
Supplement: Supplementary file 1 [file Table1.DOCX]

Supplementary Material

Spatio-temporal interdependence of bacteria and phytoplankton during a Baltic Sea spring bloom

Carina Bunse, Mireia Bertos-Fortis, Ingrid Sassenhagen, Sirje Sildever, Conny Sjöqvist, Anna Godhe, Susanna Gross, Anke Kremp, Inga Lips, Nina Lundholm, Karin Rengefors, Josefine Sefbom, Jarone Pinhassi, and Catherine Legrand*

*** Correspondence:** Corresponding Author: catherine.legrand@lnu.se

## Supplementary Tables

**Supplementary Table 1.** Environmental parameters during cruise C and D in April 2013 over the Baltic Sea for those stations where bacterial sequences are available. Salinity is provided in PSU, temperature in °C, chl*a* in relative chl*a* fluorescence, turbidity was measured in NTU, cDOM in relative fCDOM, nutrient concentrations are provided in µmol l^-1^, *S. marinoi* genotypes in % of total *S. marinoi* genotyped cells per station, phytoplankton biomass in µg C l^-1^, bacterial abundance in x10^5^ cells ml^-1^ (averaged for technical duplicates), and standard deviation for bacterial abundance in cells ml^-1^ (of technical duplicates). *S. marinoi* genotype information are reprinted from (Godhe et al., 2016).

|  | **C01** | **C02** | **C03** | **C09** | **C10** | **D01** | **D02** | **D03** | **D04** | **D05** | **D07** | **D08** | **D09** | **D10** |
| --- | --- | --- | --- | --- | --- | --- | --- | --- | --- | --- | --- | --- | --- | --- |
| salinity | 9.411 | 8.807 | 6.624 | 6.479 | 5.921 | 10.944 | 10.334 | 7.486 | 7.209 | 6.964 | 6.834 | 6.386 | 6.112 | 5.751 |
| temperature | 2.877 | 3.029 | 2.321 | 1.732 | 1.419 | 5.826 | 5.945 | 4.107 | 3.974 | 3.812 | 3.505 | 3.231 | 2.491 | 2.252 |
| chl*a* | 8.109 | 6.996 | 6.572 | 12.296 | 14.151 | 5.459 | 3.551 | 5.565 | 2.65 | 7.95 | 18.02 | 23.426 | 26.977 | 24.486 |
| turbidity | 1.212 | 1.357 | 1.018 | 1.115 | 1.163 | 0.728 | 0.824 | 1.115 | 0.728 | 0.776 | 0.873 | 0.873 | 0.97 | 0.97 |
| cDOM | 0.04 | 0.08 | 0.169 | 0.265 | 0.379 | 0.085 | 0.078 | 0.146 | 0.121 | 0.129 | 0.163 | 0.215 | 0.38 | 0.473 |
| phosphate | 0.16 | 0.23 | 0.36 | 0.42 | 0.53 | 0.04 | 0.07 | 0.08 | 0.22 | 0.28 | 0.13 | 0.09 | 0.19 | 0.28 |
| nitrate | 0.48 | 0.39 | 2.68 | 4.27 | 4.14 | 0 | 0.01 | 0.07 | 0.03 | 0.02 | 0 | 0 | 0.1 | 0.03 |
| silicate | 9.66 | 10.21 | 12.93 | 13.96 | 16.67 | 0.11 | 0.78 | 4.88 | 7.93 | 11.61 | 4.85 | 5.8 | 6.92 | 10.05 |
| *S. marinoi* genotype1 | 0.74 | 0.62 | 0.62 | 0.63 | 0.64 | 0.94 | 0.8 | 0.84 | 0.83 | 0.44 | 0.14 | 0.29 | 0.23 | 0.05 |
| *S. marinoi* genotype2 | 0.26 | 0.38 | 0.38 | 0.38 | 0.36 | 0.06 | 0.2 | 0.16 | 0.17 | 0.56 | 0.86 | 0.71 | 0.77 | 0.95 |
| *S. marinoi* cluster dominance | 1 | 1 | 1 | 1 | 1 | 1 | 1 | 1 | 1 | 2 | 2 | 2 | 2 | 2 |
| Phytoplankton biomass | 30.86 | 58.63 | 48.71 | 255.98 | 249.48 | 18.94 | 25.28 | 50.82 | 57.32 | 98.83 | 147.61 | 284.25 | 217.27 | 297.98 |
| diatoms/ dinoflagellates | 4.88 | 7.67 | 8.73 | 0.76 | 0.76 | 0.25 | 0.38 | 2.60 | 0.28 | 0.36 | 1.72 | 1.33 | 1.30 | 1.26 |
| Bacterial abundance | 18.01 | 19.02 | 16.87 | 13.59 | 11.15 | 14.02 | 22.12 | 27.84 | 18.61 | 14.81 | 10.66 | 7.9 | 10.52 | 13.18 |
| sd Bacterial abundance | 87461 | 26845 | 7679 | 62008 | 142650 | 8676 | 32155 | 147254 | 26315 | 22964 | 3133 | 7317 | 64899 | 40812 |
